# Factors Contributing to Low Adherence to Community-Based Health Insurance in Rural Nyanza District, Southern Rwanda

**DOI:** 10.1155/2018/2624591

**Published:** 2018-12-18

**Authors:** Mecthilde Mukangendo, Manasse Nzayirambaho, Regis Hitimana, Assumpta Yamuragiye

**Affiliations:** ^1^University of Rwanda, School of Health Sciences, Kigali, Rwanda; ^2^University of Rwanda, School of Public Health, Kigali, Rwanda

## Abstract

**Background:**

Community-based health insurance (CBHI) schemes are an emerging mechanism for providing financial protection against health-related poverty. In Rwanda, CBHI is being implemented across the country, and it is based on four socioeconomic categories of the “Ubudehe system”: the premiums of the first category are fully subsidized by government, the second and third category members pay 3000 frw, and the fourth category members pay 7000 frw as premium. However, low adherence of community to the scheme since 2011 has not been sufficiently studied.

**Objective:**

This study aimed at determining the factors contributing to low adherence to the CBHI in rural Nyanza district, southern Rwanda.

**Methodology:**

A cross-sectional study was conducted in nine health centers in rural Nyanza district from May 2017 to June 2017. A sample size of 495 outpatients enrolled in CBHI or not enrolled in the CBHI scheme was calculated based on 5% margin of error and a 95% confidence interval. Logistic regression was used to identify the determinants of low adherence to CBHI.

**Results:**

The study revealed that there was a significant association between long waiting time to be seen by a medical care provider and between health care service provision and low adherence to the CBHI scheme (*P* value < 0.019) (CI: 0.09107 to 0.80323). The estimates showed that premium not affordable (*P* value < 0.050) (CI: 0.94119 to 9.8788) and inconvenient model of premium payment (*P* value < 0.001) (CI: 0.16814 to 0.59828) are significantly associated with low adherence to the CBHI scheme. There was evidence that the socioeconomic status as measured by the category of Ubudehe (*P* value < 0.005) (CI: 1.4685 to 8.93406) increases low adherence to the CBHI scheme.

**Conclusion:**

This study concludes that belonging to the second category of the Ubudehe system, long waiting time to be seen by a medical care provider and between services, premium not affordable, and inconvenient model of premium payment were significant predictors of low adherence to CBHI scheme.

## 1. Background

As universal health coverage (UHC) is becoming a priority, there is a need to increase the financial accessibility of health care services, protecting the population from catastrophic expenditure, and decreasing the risk of extreme poverty [1]. The community-based health insurance (CBHI) scheme provides financial protection by reducing out-of-pocket health care spending and improves cost recovery. However, in low-income countries, low enrolment rates hamper the successful development of CBHI schemes. Low enrolment rates endanger the sustainability of CBHI schemes not only because they reduce the size of the insurance pool but also because they bear a negative impact on further enrolment and dropout [2].

Having too few people in a scheme, either because people do not enroll at all or because they do not renew their membership year after year, inevitably translates into limited resource mobilization. In turn, limited resource mobilization represents a threat for the long-term viability of schemes and the stabilization of the financial resources made available to providers, forcing schemes, which cannot cover basic costs, to suspend their operations [3].

Globally, different forms of community-based health insurance applying the principle of risk sharing were organized to provide the financial risk protection, especially for people in the poor category to ensure that no one is left behind with regard to access of health care services [4]. Current literature indicates the success in the CBHI scheme even in low- and middle-income countries [5]. However, low adhesion rate, limited resource mobilization, and poor sustainability have been the challenges to an effective implementation of the CBHI scheme in some sub-Saharan African countries [3].

While a large literature has examined factors associated with initial uptake, work on factors that determine contract renewal or prevent dropout is relatively thin [2]. The quality of care on offer, affordability of the scheme, lack of understanding of the CBHI insurance scheme, and insufficient information on how to use the insurance policy are factors that are most likely to influence renewal rates and new uptake in the CBHI scheme [6]. Low enrolment rates have been found to be related to affordability of premiums, noncooperative attitudes of health providers, poor quality of care as well as lack of basic information, and participation on the design and operations of schemes [7].

In Reference [8], while affordability is an issue, the main reason for the declining enrolment rate was the poor quality of care in Conakry, Guinea. Similarly, quality of care was perceived by household heads as an important aspect determining dropout in the case of Burkina Faso's Nouna district scheme [9]. A negative perception of quality of care increased the probability of dropping out [10]. Furthermore, [11] households with greater scheme information and better understanding of insurance were more likely to renew contracts.

Rwanda and Ghana have been successful countries in implementing the CBHI scheme in Africa [1–3]; however, the enrolment rate is getting down compared to the previous results. The perceived poor quality of care, challenge in the management of the scheme, high premium, and coinsurance are identified as factors of decrease in the enrolment rate [12]. A study conducted in Ghana has identified barriers to the enrolment rate including lack of trust in the scheme, perceived poor quality of care, long waiting time for services among insured clients, and delay in card production [12]. All of these factors are contributing to low enrolment in the developing world [13].

## 2. Rwanda CBHI Scheme

In Rwanda, community-based health insurance (CBHI) is a solidarity health insurance system in which persons (families) come together and pay contributions for the purpose of protection and receipt of medical care. It was established in order to help people with low-income access medical care at affordable cost [14]. From the 1st July, 2015, the management of the community-based health insurance scheme was moved from the Ministry of Health to the Rwanda Social Security Board. The move aimed at improving the fund's financial accountability and ensures quality health care for subscribers [15].

Members of the CBHI are entitled to a benefits package including both outpatient and inpatient care at public facilities throughout the country regardless of the place of residence. Basic care and referrals to district or tertiary hospitals are provided through the local health center. A member who benefits from medical care services has to pay a copayment of 200 frw for health center visits and 10 percent of the medical bill for hospital visits [16].

Membership is effective when each household member has, personally or through a third party, paid the required contribution. The CBHI offers health care coverage to household members if all of them have paid their respective contributions, with the exception of any member insured under other medical insurance schemes [17]. A member who joins for the first time the community-based health insurance and who pays contributions with delay starts benefiting from medical care services thirty days after the payment of his or her subscription fees. However, medical services for children aged three months and younger are covered by the contributions of their parents [14]. Note that the CBHI year starts on the 1st July and ends on the 30th June of the following year. The member has to renew his or her insurance before the benefiting year ends [16].

The CBHI scheme's revenue depends heavily on premiums collected from members at the community level and deposited on the Rwanda Social Security Board bank accounts throughout the country to ease access by depositors. Contribution is made depending on the category of the Ubudehe system to which individuals belong [17]. The Ubudehe system is a community-based targeting mechanism that categorizes the Rwandan population according to their revenues and vulnerability. The system allows Rwandese citizens and leaders at the lowest village level to analyze the existing poverty amongst their own communities in order to implement social protection programmes and better target those in need for assistance in the villages. The communities decide amongst themselves to which Ubudehe category every household in the village belongs. The major aim of the system is to ensure that the poorest segments of the population have access to health services and can receive the support needed [15, 16, 17].

Hence, based on the Ubudehe system, the premiums of CBHI Category 1 are RWF 2,000 per member and are fully subsidized by the government and development partners. Also, no copayment is charged to this category at the point of care. CBHI Categories 2 and 3 members pay RWF 3,000 per member and CBHI Category 4 members pay RWF 7,000. The user copayments described above still apply for CBHI Categories 2 and 3 members [16, 17].

Even though health care reforms in Rwanda helped to significantly increase coverage, there are still gaps in the implementation and universal coverage has not yet been reached [15]. As any other low-income country, Rwanda is still in need to increase the accessibility of health care service especially in the poorest category to ensure nobody is left behind with regard to health care services access [17]. Furthermore, enrolment rates have declined since 2011 [15]. Country wide, the enrolment rate decreased from 91.0 percent in 2011 to 74 percent and 76 percent in fiscal years 2013/2014 and 2014/2015, respectively [15].

Despite the fact that the CBHI adhesion rate lies between 70% and 80% of the total population in Rwanda and considered to be successful compared to other similar countries [1, 3, 12], the CBHI scheme in Rwanda is suffering from substantial fluctuations in membership. What make enrolled people in the CBHI scheme to not renew their membership in following years and demotivate the individual to first enroll in CBHI is still not yet sufficiently explored. Determining the factors behind the low enrolment rate is therefore an essential factor to sustain the development of CBHI in order to make health care services much more accessible to all the Rwandan population and thereby reduce the out-of-pocket expenditure contributing to poverty reduction.

This study aiming at determining factors contributing to low adherence to CBHI scheme in rural Nyanza district, southern Rwanda, presents useful insights into exploring why this is so in order to empower decision-makers with the information necessary to design measures that can enhance retention in CBHI and adherence to it; thus, increasing the sustainability of schemes in Rwanda as elsewhere in sub-Sahara Africa; yet contributing to the wealth of knowledge on the CBHI scheme.

This study was guided by the following research questions:

- Are community members satisfied with health care services provided to them?
- What is the knowledge of the CBHI scheme held by the community?
- What is the socioeconomic status of community members?
- Do community members afford CBHI scheme?

## 3. Methodology

### 3.1. Research Setting

The study was conducted in rural Nyanza district located in the south province of Rwanda, about 95 km from the capital, Kigali. Nyanza district covers an area of about 672 km^2^ and has a total population of 323,388 [17]. Nyanza district was among the bottom ten districts with the lowest health insurance coverage as reported in the Rwanda Demographic Health Survey [18]. Being below the national coverage, with its 68.3 percent of the population insured, Nyanza district was regarded as having low enrolment rate to CBHI.

### 3.2. Research Design

A quantitative approach using a descriptive cross-sectional design was used to collect data in a targeted population at a certain point in time. Data were collected by means of a pretested structured questionnaire with closed ended questions.

### 3.3. Research Sampling

The researcher used simple random sampling technique to select the study area and nine of the sixteen health centers of the rural Nyanza district. The proportionate random sampling technique was used to select the study participants among the population of selected nine health centers.

The sample for this study was all adult outpatients on exit who were members of the CBHI scheme with CBHI membership card and nonmembers of CBHI belonging to the catchment area of the selected nine different health centers across rural Nyanza district. The participants were outpatients in noncritical situation, mentally competent with age equal to or above 21 years old because at this age, a person seems to be independent and responsible, financially capable to contribute to CBHI membership.

### 3.4. Sample Size Determination

The sample size was determined by using the single population proportion formula:

(1) $$\begin{array}{c} n=\frac{1.5\;\left(Z\left(\alpha /2\right)\right)2\text{ po qo}}{d2}, \end{array}$$

where “*n*” is the sample size, “po” is the probability of success, “qo” is the probability of failure, and “*d*” is the margin of error. The design effect of 1.5 was considered because of two levels of random selection of health center and the proportionate random selection of participants in each health center. The participants were selected using the following formula:

(2) $$\begin{array}{c} \text{ni}=\frac{\text{Ni}\times n}{N}, \end{array}$$

where “ni” is the sample size proportion to be determined, “Ni” is the total population of the health center catchment area obtained from the health center coordinator, “*n*” is the sample size, and “*N*” is the total population of all randomly selected health centers. The study used the marginal of error of 5% with a significance level of 0.05. The degree of the confidence interval was 95%; Z*α*/2 = 1.96. This yielded a sample size of 499 participants.

### 3.5. Research Data Collection Tool

The researcher collected data using a questionnaire. The design and content of the questionnaire was drawn from the literature of work previously done in the area of adherence to the CBHI scheme [2, 3, 15]. The questionnaire had two parts: the first part gathered information about the participants' demographic characteristics and the last part contained questions aiming at identifying factors associated with low adherence to the CBHI scheme by determining what make enrolled people to not renew their membership in following years and demotivate the individual to first enroll in the CBHI scheme.

The questionnaire was the method used to collect information on socioeconomic status of the individual determined by the category of the Ubudehe system to which its household belongs, satisfaction with quality of health care services provided, affordability of premium and copayment fees and knowledge of the CBHI scheme management. The satisfaction with quality of health care services module includes questions regarding overall quality of health care services, availability of medical equipment and prescribed drugs, waiting time to be seen by a health care provider, waiting time between services, and health care providers' friendliness. Affordability of premium and copayment fees including the model and time of payment, ability to pay copayment, and premiums fees were recorded. The last round of the survey enquired information on whether a member attended community level CBHI meetings or training related to the scheme and whether a member had active participation in the CBHI scheme management to capture the knowledge of the scheme.

### 3.6. Research Procedure and Data Analysis

During the data collection period, the researchers went to each selected health center and randomly selected eligible study participants among outpatients on exit who came to seek health care services. The researcher used face-to-face interviews in order to avoid the exclusion of potential participants with low levels of education and unable to read and write. The selection was pursued until the required proportionate sample was reached for each selected health center and making in total a sample size of 495 participants with a response rate of 99.1 percent. The researchers as interviewers were recording in respective provided space each answer provided by each participant.

Data collected were entered into the computer software program StataSE13 for statistical analysis. Descriptive statistics, bivariate, and multiple logistic regression analyses were performed. The *P* value of 0.05 and 95% confidence intervals were used to determine associations between independent and dependent variables. Tables were used to present findings.

### 3.7. Research Variables

Adherence to CBHI was binary variable, nonadherence versus adherence. We treated the probability that a person does not adhere to the CBHI scheme as a function of a range of factors that are likely to influence the no demand for health insurance. In particular, we focused on the role of four main sets of variables, that are, socioeconomic status of the individual determined by the category of the Ubudehe system to which its household belongs, satisfaction with quality of health care services on offer, affordability of premium and copayment fees, and knowledge of the CBHI scheme, in determining low adherence to the CBHI scheme.

### 3.8. Ethical Consideration

Ethical clearance to conduct the study was obtained from the Institutional Review Board of the University of Rwanda, College of Medicine and Health Sciences. Permission to carry out the study was granted by the management of Nyanza district. The researchers explained the purpose and objectives of the study to the participants before the completeness of the questionnaire. A consent form was given to study participants to inform them about the voluntary participation to the study, their right to withdraw from the study at any time without penalty, participants' right to freedom of choice and expression, and the anonymity and confidentiality during the study.

## 4. Results

### 4.1. Demographic Characteristics of Respondents

Table 1 shows the demographic characteristics of respondents. Of the total respondents, 79.2% were females and 87.9% were aged between 21 and 60 years. 338 (68.3%) were married. Occupationally, 89.5% of the respondents were laborers. Regarding educational status, 63.84% of the respondents could read and write and 36.16% were illiterate. With respect to Ubudehe categorization, 22% of the respondents were in the Ubudehe category, one fully subsidized by the government and 32.1% and 45.9% were in Categories 2 and 3, respectively.

### 4.2. Socioeconomic Status of Respondents

Table 2 reports bivariate estimates of association between adherence and socioeconomic status of individual measured by the Ubudehe categorization system of the household in which respondent falls. The estimate shows that respondents who belong to the second category (*P* value < 0.004) (CI: 0.13379 to 0.68361) of the Ubudehe system are likely to not adhere to the CBHI scheme.

### 4.3. Satisfaction with Quality of Health Care Services on Offer

The set of estimates reported in Table 3 shows that there is linear relation between adherence to CBHI scheme and dissatisfaction of outpatient on the exit in regard to long waiting time to be seen by a medical care provider (*P* value < 0.038) (CI: 0.05975 to 0.92132). For example, he/she has to wait one to three hours to be seen by the health care provider (*P* value < 0.057) (CI: 0.114523 to 1.03183) and approximately one hour between services (*P* value < 0.055) (CI: 0.170098 to 1.01988). In contrast, satisfaction status of overall quality of services, availability of drugs, and diagnostic facilities do not influence low adherence to the CBHI scheme. Moreover, cleanliness of the facility and friendliness of staff were found to not be statistically signiﬁcant.

### 4.4. CBHI Scheme Affordability

The set of estimates from the bivariate analysis presented in Table 4 shows that premium not affordable (*P* value < 0.053) (CI: 0.05245 to 1.01877) and inconvenient model of premium payment (*P* value < 0.004) (CI: 1.29623 to 3.90412) are significantly associated with low adherence to the CBHI scheme. In contrast, there is no statistically significant relation between adherence and copayment affordability.

### 4.5. Knowledge of the CBHI Scheme

Of the two variables included to capture scheme-specific knowledge, no clearest effect emerges from the meetings attended to discuss about waiting time to use services after premium payment and the participation to the scheme management with adherence to the CBHI scheme. The estimates in Table 5 from bivariate analysis confirm no statistical signiﬁcance of the two variables with low adherence to the CBHI scheme, and a joint test for the statistical significance of the two dummy variables records a *P* value of 0.98.

### 4.6. Predictors of Low Adherence to CBHI Scheme in Rural Nyanza District, Southern Rwanda

Table 6 shows that in the multivariate analysis, almost all the explanatory variables had the hypothesized sign. The following factors all had an effect on low adherence, meaning that they increased the probability of low adherence to the CBHI scheme: premiums not affordable, being a member of a household in the second category of the Ubudehe system, perceived long waiting time to be seen by a medical care provider and between services and inconvenient model of premium payment. However, scheme knowledge, availability of diagnostic facilities, and copayment fees affordability did not have the hypothesized sign.

Accordingly, respondents belonging to the second category of the Ubudehe system were likely not to adhere to the scheme as the estimate is statistically significant (*P* value < 0.005) (CI: 1.4685 to 8.93406). Similarly, there is evidence that a negative perception of the quality of health care such as long waiting time to be seen by a medical care provider (*P* value < 0.019) (CI: 0.09107 to 0.80323): one hour to three hours were required for the same (*P* value < 0.034]) (CI: 0.09536 to 0.91306) and at least one hour between services (*P* value < 0.047) (CI: 0.16505 to 0.99003) influence low adherence to the scheme. In addition, unaffordable premiums (*P* value < 0.050) (CI: 0.94119 to 19.8788) and inconvenient model of premium payment (failure to raise premiums for all members of the household before enrolment) (*P* value < 0.001) (CI: 0.16814 to 0.59828) were statistically associated with low adherence to the CBHI scheme.

## 5. Discussion

The findings of our study show the second category of the Ubudehe system (the system assigns households in which the participant falls into one of the four categories, based on their income and assets) stated as socioeconomic factor revolving around the low adherence to the CBHI scheme. This category pays a slightly higher premium of RWF 3,000 by a person as they are considered to be wealthier compared to ones in the first category fully subsidized by the Government [14]. This is similar to what reported by the International Labor Office, where low enrolment rates to the CBHI scheme were highlighted, and it was partly due to incorrect categorization of members in the Ubudehe system. Some members are categorized as wealthier than they actually are and tend to not adhere. Other members, whilst correctly classified, experience difficulties in paying the premiums due to seasonal or irregular incomes [19].

Among respondents, members of the CBHI scheme, 33.54% respondents and 56.33% respondents in category two and three, respectively, do not plan to renew their CBHI membership. Surprisingly, 10.13% respondents in the first category do not plan their membership renew (Table 7). This is in line with other study conducted in Nigeria which reported the same in terms of wealth quintile members and enrolment, whereby those with high income were less likely to adhere than those with lower income [20]. Moreover, in a study conducted in Nouna, Burkina Faso, it was found that the individual of higher socioeconomic status was positively correlated with low adherence to the CBHI scheme [9].

The findings from this study reveal that the poor quality of health care is another key factor to influence the low adherence to the CBHI scheme. The results of our multivariate analysis confirmed the association between low enrolment to CBHI and long waiting time to be seen by a medical care provider and between services. These findings are in line with other studies, where scholars revealed that long waiting times have been criticized by respondents as one issue that affect adherence to the CBHI scheme in Burkina Faso [7]. Similar findings were reported in one study conducted in Builsa district of Ghana, where participants criticized waiting times to be too long for health services delivered [21]. Other studies conducted in Burkina Faso [9] and Nigeria [22] linked the quality of health care and low enrolment to the CBHI scheme in the sense that individuals that perceived quality of care as good were found to enroll than those who perceived the quality with less admiration. Furthermore, a study conducted in Conakry, Guinea pointed to the poor quality of care in the health care services as one of the main causes of the low and even declining enrolment in CBHI despite initial enthusiasm at the setup of the CBHI scheme [8].

The findings of our study point out the inconvenient model of premium payment (difficulties in raising premiums for all members before enrolment) as major factors of low adherence to the CBHI scheme. The findings are similar to the ones in the CBHI-Ishaka scheme in Uganda, where such requirements were measures against adverse selection [23]. One study conducted in Burkina Faso [7] revealed that institutional rigidities in payment modality were found to contribute to low adherence to the CBHI scheme. For instance, the participants stressed that a single payment is more problematic in rural areas, where it is hard to obtain credit as incomes of workers in the informal or agricultural sectors vary over the course of the year and premium may due at a time of year when their financial situation is poor [7, 24].

The findings of this study indicate unaffordable premium as significantly contributing to low adherence to the CBHI scheme. This situation is attributable to low levels of income or lack of financial resources. As shown in Table 8, more than 85% of the respondents and nonmembers of the CBHI scheme mentioned premium not affordable as reason to not adhere to CBHI. Furthermore, 37.44% respondents, members of CBHI conform to not renew their membership (Table 9) because they cannot afford regular premiums (Table 10). These are in line with what reported in study conducted in Uganda, where inability to pay for membership was pointed out as the foremost reason for not joining the scheme. The reason most mentioned by research participants for not joining the scheme was lack of money and being unable to pay contributions for their large families [23]. Similarly, inability to pay the premium as the main reason for low adherence to the scheme was reported in a study conducted in Ethiopia [2]. Moreover, poverty was identified as a key barrier to enrolment in Burkina Faso, where the authorities used community wealth ranking to identify the poorest households, who then received significant subsidies [24].

The findings of this study show that there is no linear relation between knowledge of the CBHI scheme and low adherence. The good knowledge of the CBHI scheme is likely to be due to successful awareness-raising activities that convey information in an effective manner. However, it must be observed that a good understanding of CBHI principles, per se, will not directly translate into increased enrolment. These findings are in line with another study conducted in the Maliando Mutual Health Organisation which indicated that the enrolment rate was low, despite good understanding of the concepts and principles of the scheme [8].

### 5.1. Methodological Considerations

The use of individual survey data (outpatients on the exit) rather than household survey data represents an important limitation of our study. However, this optional may exclude the skewed image brought in by household as unit of analysis on individual adhesions levels. In addition, we must acknowledge that the study was conducted only with outpatients on the exit, which may limit generalizing the results. The sample may not represent those who did not come to the health facilities.

A potential criticism could be directed against our decision to recruit our participants in health facilities. It was likely that we got higher percentage of people with insurance than the general population and more female respondents than men because there is a proportion of noninsured who did not come to health facilities. Furthermore, the cross-sectional as the quantitative study design used has a basic limitation in assessing the direction of causality between the outcome and exposure. Only relationships and associations have been deduced for this study.

Revealing the reasons behind identified factors that contribute to low adherence to the CBHI scheme, however, is beyond the reach of quantitative analysis relying on individual survey data and requires a complementary qualitative method of analysis to explore in greater detail the set of factors identified, but not easily explained, by regression modelling.

## 6. Conclusion

This study aimed at determining factors contributing to low adherence to the CBHI scheme in rural Nyanza district, southern Rwanda. The study concludes that there is the potential link between low adherence to the scheme and belonging to the second category of the Ubudehe system, long waiting time to be seen by a medical care provider and between services, premium not affordable by the community, and inconvenient model of premium payment (failure to raise premium for all members of the household before enrolment). The highlighted factors contribute highly to low adherence to the CBHI scheme.

The study recommends a review of guidelines of community wealth ranking which could lead to recategorization into the Ubudehe system and a revisit of premium collection guidelines and modalities and makes them convenient to individuals and household in order to reduce low adherence to CBHI.

## Figures and Tables

**Table 1:** Sociodemographic characteristics of respondents.

Variables	Frequency	%
*Sex of patient*		
Male	103	20.8
Female	392	79.2

*Age in Category*		
21–40 years	306	61.8
41–60 years	129	26.1
61+ years	60	12.1

*Marital Status*		
Single	75	15.2
Married	338	68.3
Widowed	63	12.7
Divorced	19	3.8

*Occupation*		
Laborer	443	89.5
Housewife	13	2.6
Merchant	6	1.2
Student	8	1.6
Others	25	5.1

*Education*		
Illiterate	179	36.16
Read and write	281	56.77
Secondary school	32	6.46
Tertiary school	3	0.61

*Ubudehe Category*		
Category 1	109	22
Category 2	159	32.1
Category 3	227	45.9
*Total*	495	100

**Table 2:** Socioeconomic status related determinant of low adherence to CBHI scheme.

Adherence to CBHI scheme	Standard error	*P* value	95% confidence interval	
*Ubudehe Category*				
Category *2*	0.125842	<0.004	0.13379	0.68361
Category *3*	0.199759	0.078	0.21447	1.08625
_Cons.	4.63702	0	6.14603	25.9339

**Table 3:** Satisfaction with quality of health care services on offer-related determinant of low adherence to CBHI scheme.

Satisfaction with quality of health care services on offer	Adherence to CBHI scheme			
	Standard error	*P* value	95% confidence interval	
*Overall quality of services*				
Not satisfied	1.139123	0.886	0.1659264	8.00003
Satisfied	1.147767	0.655	0.2972427	6.89163

*Availability of Drugs*				
Not satisfied	0.59327	0.787	0.2005757	3.38001
Satisfied	0.373491	0.414	0.1787233	2.03063

*Availability of diagnostic facilities*				
Not satisfied	0.264296	0.165	0.0726466	1.56657
Satisfied	0.294097	0.224	0.1301865	1.6122

*Cleanliness of the facility*				
Not satisfied	8.260412	0.099	0.6915176	70.7773
Satisfied	5.197502	0.179	0.4975695	42.2756

*Waiting time to see medical provider*				
Not satisfied	0.16374	<0.038	0.0597501	0.92132
Satisfied	0.683126	0.688	0.0868455	5.01757

*Waiting time between services*				
Not satisfied	0.879014	0.891	0.1203727	6.29799
Satisfied	0.594289	0.901	0.2613374	3.26028

*Friendliness of staff*				
Not satisfied	0.56661	0.658	0.1421747	3.42643
Satisfied	0.685693	0.992	0.2650326	3.82519

*Time waited to see medical provider*				
30 to 60 minutes	0.40087	0.83	0.3836129	2.15773
1 to 3 hours	0.192779	0.057	0.1145227	1.03183
3 to 6 hours	0.281293	0.203	0.1362349	1.5276
6 hours and more	0.574392	0.781	0.2099668	3.23107

*Time waited between services*				
30 to 60 minutes	0.190308	0.055	0.1700983	1.01988
1 to 3 hours	0.262326	0.173	0.1497689	1.40697
3 to 6 hours	0.502994	0.663	0.1987046	2.79737
6 hours and more	0.336043	0.3	0.1304898	1.8734
_Cons	0.789734	0.662	0.0233549	10.8729

**Table 4:** CBHI scheme affordability-related determinant of low adherence to CBHI scheme.

Scheme affordability	Adherence to CBHI scheme			
	Standard error	*P* value	95% confidence interval	
*Premium payment model*				
Convenient	0.75586	0.684	0.39771	4.07595
Not convenient	0.63275	<0.004	1.29623	3.90412

*Copayment fees*				
Affordable	0.84025	0.568	0.43637	4.53558
Not affordable	0.3345	0.888	0.55885	1.95762

*Premium fee*				
Affordable	0.83018	0.737	0.05387	7.90112
Not affordable	0.17493	0.053	0.05245	1.01877
_Cons	9.77195	0.001	2.81992	57.3424

**Table 5:** Knowledge of the CBHI scheme-related determinant of low adherence BHI scheme.

Adherence to CBHI scheme	Standard error	*P* value	95% confidence interval	
*Meeting and Training on CBHI*				
Not attended	0.593977	0.561	0.0500329	5.0769
Attended	0.606072	0.575	0.0530903	5.101125
_Cons	0.384903	0.341	0.0346738	3.204521

*Participate in CBHI management*				
No participation	0.319795	0.977	0.5261158	1.865029
Participated	0.412219	0.413	0.6957266	2.418136
_Cons	0.038206	0	0.0990319	0.254416

**Table 6:** Predictors of low adherence to CBHI scheme in rural Nyanza district, 2017.

Adherence to CBHI scheme	Standard error	*P* value	95% confidence interval	
*Ubudehe category*				
Category 2	1.66845	<0.005	1.4685	8.93406
Category 3	0.79984	0.215	0.72014	4.28817

*Availability of diagnostic facilities*				
Not satisfied	0.1878	0.062	0.06349	1.06995
Satisfied	0.24524	0.134	0.11239	1.33924

*Waiting time to see medical provider*				
Not satisfied	0.15021	<0.019	0.09107	0.80323
Satisfied	0.35732	0.408	0.20165	1.91777

*Time waited to see medical provider*				
30 to 60 minutes	0.41139	0.870	0.39085	2.21322
1 to 3 hours	0.17006	<0.034	0.09536	0.91306
3 to 6 hours	0.269	0.175	0.11718	1.47737
6 hours and more	0.48247	0.597	0.1764	2.71392

*Time waited between services*				
30 to 60 minutes	0.18474	0.047	0.16505	0.99003
1 to 3 hours	0.22965	0.110	0.12608	1.23469
3 to 6 hours	0.52811	0.714	0.20692	2.94045
6 hours and more	0.53395	0.703	0.19573	3.0023

*Premium payment model*				
convenient	0.40003	0.456	0.17263	2.19946
Not convenient	0.1027	<0.000	0.16814	0.59828

*Copayment fees*				
Affordable	0.38021	0.418	0.17151	2.07982
Not affordable	0.29631	0.651	0.43358	1.68648

*Premium fees*				
Affordable	3.01025	0.546	0.16236	31.0667
Not affordable	3.36583	<0.050	0.94119	19.8788
_Cons	0.69819	0.667	0.06517	5.73393

**Table 7:** Renew status of CBHI membership by category of Ubudehe

Ubudehe category	Renew of CBHI membership		
	Yes	No	Total
Category 1	85	16	101
	32.25%	10.13%	23.93%

Category 2	73	53	126
	27.65%	33.54%	29.86%

Category 3	106	89	195
	40.15%	56.33%	46.21%

Total	264	158	422
	100	100	100

**Table 8:** Reasons to not adhere to CBHI scheme.

Reasons to not adhere to CBHI scheme	Adherence to CBHI scheme	
	No (*N* = 73)	Total
Illness and injury not frequent	7	7
	8.77%	9.72%

Premium not affordable	61	61
	85.92%	84.72%

Wait and see the benefits	3	3
	4.23%	4.17%

Other reasons	1	1
	1.41%	1.39%

Total	72	72
	100	100

**Table 9:** Renew status of CBHI membership.

Renew of CBHI membership	Adherence to CBHI scheme	
	Yes	Total
Yes	264	264
	62.56%	62.56%

No	158	158
	37.44%	37.44%

Total	422	422
	100	100

**Table 10:** Reasons for not renewing CBHI membership.

Reasons for not renewing CBHI membership	Renew of CBHI membership	
	No	Total
Illness and injury no frequent	4	4
	2.55%	2.55%

Premium not affordable	153	153
	97.45%	97.45%

Total	157	157
	100	100

## Data Availability

The data used to support the findings of this study are available from the corresponding author upon request.
